# Comparison of Intraocular Pressure Measurements with Goldmann Applanation Tonometry, iCare, and Tono-Pen in Young Children with Anterior Segment Abnormalities Under General Anesthesia

**DOI:** 10.3390/jcm14103338

**Published:** 2025-05-11

**Authors:** Matias K. Studer, Milko Iliev, Christoph Tappeiner, Beatrice E. Frueh, Stephan A. Fraenkl

**Affiliations:** 1 Department of Ophthalmology, Inselspital, Bern University Hospital, University of Bern, 3010 Bern, Switzerland; matias.klaus.studer@gmail.com (M.K.S.); miliev44@yahoo.de (M.I.); beatrice.frueh@insel.ch (B.E.F.); 2 Department of Ophthalmology, Pallas Kliniken, 4600 Olten, Switzerland; christoph.tappeiner@pallas-kliniken.ch; 3 Department of Ophthalmology, University Hospital Essen, University Duisburg-Essen, 45147 Essen, Germany; 4 Medical Faculty, University of Bern, 3008 Bern, Switzerland

**Keywords:** intraocular pressure, childhood glaucoma, rebound tonometer

## Abstract

**Background**: In young patients with suspected elevated intraocular pressure (IOP), examinations under general anesthesia remain the gold standard. This study aimed to compare the reliability of Goldmann applanation tonometry (Perkins), iCare rebound tonometry, and the Tono-Pen in young children under general anesthesia in a clinical setting. **Methods**: This retrospective study included patients under six years of age requiring an ophthalmic examination under general anesthesia. IOP measurements were performed using all three devices, and central corneal thickness (CCT) was recorded for each patient. **Results**: A total of 38 eyes of 19 children (mean age, 1.8 ± 2.1 years) were included. IOP values of all three devices ranged from 5 to 43 mmHg, with a mean CCT of 645.6 ± 135 µm. The Tono-Pen recorded significantly higher IOP values than the Perkins tonometer (15.2 ± 5.5 mmHg vs. 11.1 ± 4.8 mmHg; *p* = 0.002), while no significant differences were observed between Perkins and iCare. CCT was significantly correlated with iCare (r = 0.344, *p* = 0.032) and the Tono-Pen (r = 0.519, *p* = 0.001) but not with Perkins (r = 0.247, *p* = 0.129). Bland–Altman analysis showed a significant slope for inter-device differences, but when excluding IOP values >25 mmHg, the slope was no longer significant. **Conclusions**: Among the devices tested, the Perkins tonometer was the least affected by other parameters such as CCT and IOP values in young patients under general anesthesia, particularly when IOP exceeded 25 mmHg or corneal thickness was increased. In patients with normal corneas and IOP below 25 mmHg, iCare provided comparable accuracy to Perkins, while the Tono-Pen consistently overestimated IOP compared to both devices.

## 1. Introduction

The accurate measurement of intraocular pressure (IOP) is essential for the diagnosis and management of glaucoma and other ocular conditions [1,2]. Goldmann applanation tonometry (GAT) is widely regarded as the gold standard for IOP measurement in adults due to its high accuracy and reliability [1]. However, IOP measurement in children presents unique challenges, particularly in cases where patient cooperation is limited. Young children, especially those with congenital or developmental eye conditions, often require general anesthesia for precise IOP assessment, making it necessary to use handheld tonometers that can function effectively in this setting [3]. Congenital glaucoma, a rare but serious disease with an incidence of 1 per 10,000 to 20,000 live births, is one of the key conditions that necessitates IOP measurement in young children [4]. Left undiagnosed or untreated, congenital glaucoma can lead to irreversible visual impairment due to optic nerve damage caused by sustained elevated IOP [5]. Monitoring IOP in children with this condition is crucial for timely intervention, including surgical procedures such as trabeculotomy or goniotomy. In cases of anterior segment abnormalities or post-surgical follow-up, repeated IOP assessments under general anesthesia may be required [6]. Traditionally, GAT remains the gold standard for IOP measurement, but it has practical limitations in pediatric patients [7]. Since it requires a slit lamp setup and patient cooperation, its use in young children is often impractical. Therefore, the portable version of GAT, the Perkins tonometer, has been developed and has been used as a gold standard device to measure IOP in lying patients for many years [8]. It has been shown that GAT and Perkins have a very good accordance [3]. But Perkins needs a skilled examiner and is sometime difficult to perform in irregular corneas [9]. As a result, handheld tonometers such as the Tono-Pen and iCare PRO have been developed to facilitate IOP measurement in children, including those under general anesthesia [10]. Such measurements are feasible even in very young infants [11]. These devices offer portability, ease of use, and the ability to obtain measurements in supine or non-cooperative patients, making them valuable alternatives to GAT in pediatric settings.

The Tono-Pen (Tono-Pen^®^ XL, Reichert Technologies, Depew, NY, USA) was introduced in 1987 and has been widely used for pediatric IOP assessment. This device operates on strain gauge technology, where a microsensor detects changes in corneal resistance during applanation [12]. The Tono-Pen is particularly useful for bedridden patients and those under general anesthesia, as it allows for easy one-handed operation while the patient remains in a supine position. Despite its advantages, concerns have been raised about its accuracy in comparison to GAT, with some studies indicating a tendency for the Tono-Pen to slightly overestimate or underestimate IOP depending on corneal properties [13,14].

More recently, rebound tonometry has gained popularity with the introduction of the iCare PRO and other, similar devices. Unlike applanation-based methods, rebound tonometry measures IOP by analyzing the deceleration of a small probe that makes brief contact with the cornea. This method eliminates the need for topical anesthesia [15] and is well-tolerated by children. The iCare PRO and iCare ic200 models are specifically designed for use in supine patients, making them suitable for IOP measurement under general anesthesia. The inbuilt sensor records the movement of a propelled probe and calculates the IOP by the deceleration of the probe after contact with the cornea [10,16]. It is the iCare PRO that is used most frequently in published studies and that demonstrates good concordance with GAT in healthy individuals and in patients with glaucomatous disease [17,18]. Studies have shown that the iCare PRO demonstrates good agreement with GAT in both healthy individuals and glaucoma patients, although differences in corneal biomechanics can affect the readings [19]. A key challenge in device comparisons for pediatric IOP measurement is the influence of the biomechanical properties of the cornea, especially in young children with congenital glaucoma or anterior segment anomalies [20]. In addition to corneal biomechanics, central corneal thickness (CCT) and other factors have been shown to affect IOP readings obtained with different tonometers [19]. Perkins, the Tono-Pen, and iCare all measure IOP differently, and their readings can vary depending on corneal rigidity and thickness [13]. For instance, the Tono-Pen and iCare seem to be more influenced by corneal biomechanics compared to Perkins, which can lead to slight variations in measurements [17].

The purpose of this study is to compare three clinically used devices—Perkins, the Tono-Pen, and the iCare PRO—for measuring IOP in young children under general anesthesia in a routine clinical setting, to assess the agreement between these tonometers and to identify potential systematic differences. This will help ophthalmologists make informed decisions when selecting a tonometer for use in children, particularly those undergoing surgery or requiring frequent IOP monitoring under anesthesia.

## 2. Materials and Methods

### 2.1. Study Population

In this retrospective study, data were collected from pediatric patients that underwent intraocular pressure measurements under general anesthesia at the Department of Ophthalmology, Bern University Hospital, Bern, Switzerland. The inclusion criteria were age ≤6 years, the indication for ophthalmological examination and IOP measurement under general anesthesia, and clear corneas without scars or evident leucoma. Haab’s Striae were not an exclusion criterion. The decision to perform general anesthesia was made by the pediatric ophthalmologist based on clinical necessity—for example, to obtain accurate refraction or reliable IOP measurements in non-cooperative children, or when other diagnostic procedures could not be performed adequately in an awake state. Patients underwent general anesthesia with sevoflurane. Induction of anesthesia was carried out under propofol with sevoflurane 8% in 100% oxygen carrier gas, with maintenance under spontaneous ventilation. Airway maintenance devices were inserted. Afterwards, a drop of oxybuprocaine 0.4% was applied, and IOP measurements were performed as soon as the anesthesia was effective. IOP measurements were initiated within the first minute after topical anesthesia became effective and were completed within five minutes. All IOP measurements were consistently performed by the same examiner.

### 2.2. Devices and Measurement Methods

The first device used was the iCARE PRO (iCare Finland Oy, Vantaa, Finland). Six consecutive measurements were taken in the right eye. In the case of an acceptable reading (deviation within normal limits as indicated by the device), the same procedure was performed in the left eye. In the case of a larger deviation (deviation outside normal limits as indicated by the device (<15% deviation)) in readings, six new measurements were taken until an acceptable reading was obtained.

The second device used was the Tono-Pen (Reichert Technologies, Depew, NY, USA). First, it had to be calibrated. Four consecutive measurements were taken in the right eye. In the case of a good reliability (deviation < 5%), the same procedure was performed in the left eye. In the case of a high deviation in the readings, the measurement was repeated until a good reliability was obtained.

The third device used was the Perkins tonometer (Haag Streit, Köniz, Switzerland). Fluorescein (BioGlo Fluorescein Sodium strips, HUB Pharmaceuticals, Rancho Cucamonga, Canada) was applied on the tear film and IOP measurement was performed first on the right, and then on the left eye (corresponding to a Goldmann applanation tonometry reading). Care was taken on the proper lubrication of the eyes.

The reason for this sequential testing was to minimize the trauma to the corneal epithelium (for example, after the application of fluorescein and (repeated) applanation with the Perkins tonometer) and its influence on the following exams. The probe of the iCare Pro instrument is the smallest of all three devices, and the physical stress to the cornea is the lowest.

After the IOP readings, central corneal thickness (CCT), corneal diameter, and axial length were measured with an Ultrasonic B scanner UD-8000 (TOMEY, Nagoya, Japan), objective refraction was obtained (Heine Beta 200 Skiaskop, HEINE Optotechnik GmbH, Herrsching, Germany) and gonioscopy and handheld slit-lamp examination (Kowa SL-17, portable slit lamp, Torrance, CA, USA) were performed.

### 2.3. Statistical Analysis

The comparison of IOP measurements with the three devices was analyzed using the Kruskal–Wallis test followed by the Dunn’s post hoc test. A Williams-T-Test was performed to analyze dependent correlations between devices and pachymetry. The Bland–Altmann test was performed to visualize the dependence of two methods and the dependence of readings from pachymetry. This analysis was made for all existing IOP measurements.

In a second step, the analysis was performed a second time by excluding measurements above 25 mmHg in order to evaluate the effect of high IOP readings on the accuracy of the methods. Lines indicate the mean of the differences and +/− two standard deviations (lower, upper limits of agreement). A critical difference indicates two standard deviations. Regression results were reported as slopes with 95% confidence intervals and *p*-values. Slopes were calculated using a linear mixed-effects model. Data analysis was performed using GraphPad Prism Software (version 9.5.1) and the statistical software R (version 3.5.0).

This study was approved by the ethical committee of Bern, Switzerland (BASEC No. 2020-00635) and written informed consent was obtained from all children’s parents.

## 3. Results

A total of 19 children (38 eyes) were included in this study (female n = 11, 58%). Patients were between one month and six years old (mean age 1.8 ± 2.1 years). A total of 18 eyes were aphakic, 10 eyes had a congenital cataract, 7 eyes had congenital glaucoma (1 of them was also aphakic), 3 had a nystagmus and/or hyperopia, and 2 fellow eyes were normal (Table 1). IOP ranged between 5 mmHg and 43 mmHg (iCare: 6–43 mmHg; Tono-Pen: 5–37 mmHg; Perkins: 5–34 mmHg).

Pachymetry was 646 µm ± 135. The corneal diameter measured 10.6 ± 1.4 mm horizontally and 10.1 ± 1.5 mm vertically. Comparing the three devices, the only statistically significant difference between groups was the Tono-Pen compared with the Perkins tonometer, i.e., the Tono-Pen results were higher than the Perkins results (15.2 ± 5.5 mmHg vs. 11.1 ± 4.8 mmHg; *p* = 0.02). The iCare values were slightly higher than those measured with Perkins as well, but this finding was not statistically significant (11.6 mmHg ± 4.4 vs. 11.1 ± 4.8 mmHg; *p* > 0.05).

The best correspondence was between iCare and Perkins. As the measures strongly deviated from normal distributions as evaluated in Q-Q plots, Spearman correlations were calculated. Pachymetric measures were significantly correlated with iCare (*r* = 0.344, *p* = 0.032) and the Tono-Pen (*r* = 0.519, *p* = 0.001) but not Perkins (*r* = 0.247, *p* = 0.129).

There was a significant correlation between the difference between Perkins and the Tono-Pen and pachymetry (*p* = 0.022). The thicker the cornea, the larger the difference between the Tono-Pen and Perkins.

A Williams-T-Test for comparing dependent correlations revealed that the correlation between pachymetry and the Tono-Pen was not significantly stronger than the one between pachymetry and iCare (*t*(36) = −1.40, *p* = 0.170), but it was significantly stronger than the correlation between pachymetry and Perkins (*t*(36) = −2.30, *p* = 0.027).

**Figure 1:**
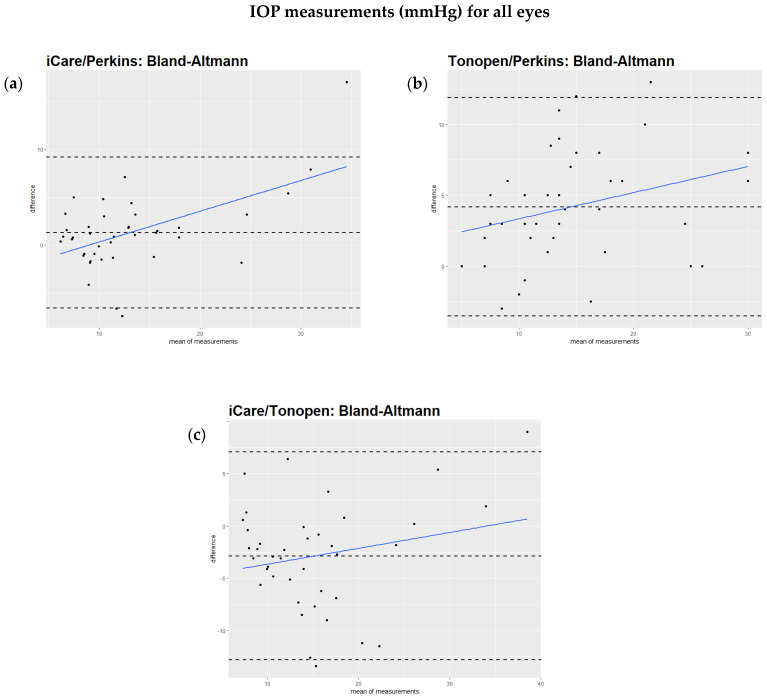
(**a–c**)**:** Bland–Altmann analysis of intraocular pressure readings comparing different devices. All values are described in mmHg. (**a**): Bland–Altmann analysis comparing iCare and Perkins readings. The slope is positive (0.42). The result is highly significant (*p* < 0.005). (**b**): Analysis comparing Tono-Pen and Perkins readings. The slope is positive (0.2). The result is significant (*p* = 0.04). (**c**): Bland–Altmann analysis comparing iCare and Tono-Pen readings. The slope is positive (0.25). The *p* value is 0.02.

**Figure 2:**
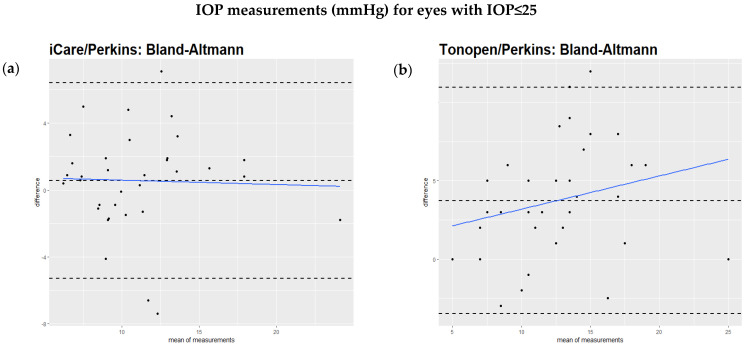
(**a**,**b**)**:** Bland–Altmann analysis of intraocular pressure readings comparing different devices excluding measurements above 25 mmHg. All values are described in mmHg. (**a**)**:** Bland–Altmann analysis comparing iCare and Perkins readings excluding measurements above 25 mmHg. When excluding readings above 25 mmHg, no significant difference between the two methods was found (regression slope = −0.12, *p* = 0.39). (**b**)**:** Analysis comparing Tono-Pen and Perkins readings excluding measurements above 25 mmHg. When excluding readings above 25 mmHg, no significant difference was found between the two devices (regression slope = 0.2, *p* = 0.24).

The Bland–Altmann test showed that the regression of the difference against the mean of the two methods had a significant slope, as can be seen in the comparison of iCare to Perkins (Figure 1a), the Tono-Pen to Perkins (Figure 1b), and iCare to the Tono-Pen (Figure 1c). However, when excluding IOP values over 25 mmHg, the regression of the difference against the mean of the two methods did not show a significant slope (Figure 2a,b). This relationship is also illustrated by scatterplots showing the IOP readings from two devices for each study eye separately (Supplementary Figure S1a–c). Table 2 summarizes these findings.

## 4. Discussion

Measurement of the intraocular pressure with GAT is considered the gold standard. The iCare tonometer is based on the method of “rebound tonometry”. The iCare tonometer enables non-invasive and well-tolerated IOP measurements in children without the need for topical anesthesia, making it particularly suitable for non-cooperative pediatric patients. Its implementation has substantially reduced the frequency of examinations under general anesthesia, especially for routine screening. These findings are in agreement with the study by Grigorian et al., which demonstrated a significant reduction in the number of examinations under anesthesia following the introduction of iCare [18].

The current study showed a good correlation between the rebound tonometer iCare and Perkins applanation handheld tonometer. This finding aligns with other studies reporting a good correlation between iCare and Perkins in children [21]. Borrego Sanz et al. reported a difference between these two tonometers in children with primary congenital glaucoma of 0.42 ± 3.69 mmHg, with higher iCare readings [22]. In another study of Martinez-de-la-Casa et al., the comparison between Perkins and iCare in congenital glaucoma also showed a good correlation with significantly higher readings in iCare (mean of 3.1 mmHg) [23].

In pediatric glaucoma with different corneal pathologies, Angmo et al. found a mean difference of 0.82 mmHg [24], and Umfress et al. found a mean difference of 1.2 mmHg [25]. Stoddard-Bennett et al. described in healthy children a mean difference of 0.72 mmHg with no influence of central corneal thickness [26].

Lambert et al. described in a review a good correlation between rebound tonometry and Goldman applanation tonometry with a higher reading in rebound tonometry of 2 to 3 mmHg higher in the 2 level II studies performed in a clinic setting and in 1 level III study performed on children under general anesthesia [21].

In our study, which included a variety of diagnoses and a higher number of aphakic eyes, no significant difference was observed between the iCare and the Perkins device (11.6 mmHg ± 4.4 vs. 11.1 ± 4.8 mmHg). This is noteworthy, as the mean corneal pachymetry in our cohort (mean 645.6 µm ± 135.4) was higher than in other studies, such as that by Martinez-de-la-Casa et al. (556.5 ± 56.1 µm) [23]. Higher IOP readings with iCare in thicker corneas have been documented in the literature [27], a finding confirmed in our study. Nevertheless, the difference between iCare and Perkins was small in this study. Esmael et al. reported a small but statistically significant difference of −0.59 ± 2.59 mmHg between rebound and applanation tonometry in children [28]. In our study, the mean difference was of similar magnitude (−0.5 mmHg), although it did not reach statistical significance. This may be attributed to the smaller sample size in our cohort. Moreover, while Esmael et al. observed declining agreement above 15 mmHg, our data indicate a more pronounced divergence above 25 mmHg, supporting the recommendation to confirm elevated IOP with Perkins applanation tonometry in clinical practice.

The thicker central corneal thickness in this study might be explained by the fact that a large number of patients with anterior segment abnormalities, notably aphakia, have been included. It has been shown that patients with aphakia and small cornea diameters have thicker corneas and therefore have a greater-than-normal measured IOP [29].

Consistent with the findings of Yulia et al. [30], our study confirms that rebound tonometry is a valuable tool for IOP screening in pediatric populations. Both studies reported an increasing discrepancy between rebound and applanation tonometry at higher IOP levels—above 25 mmHg in our cohort and above 19 mmHg in theirs. However, unlike Yulia et al. [30] who observed a tendency of rebound tonometry to overestimate IOP in children with congenital corneal opacities, we cannot evaluate this effect, as corneal opacity was an exclusion criterion in our study.

When we excluded IOP readings above 25 mmHg, the differences between the methods decreased. The slope of regression of the differences was no longer significant. The same applied to the regression of differences against pachymetry. A substantially higher deviation between devices in IOP readings above 22 mmHg has also been shown by others [31].

In this study, IOP readings were obtained under general anesthesia. It is known that anesthetic agents can alter IOP readings in a time-dependent manner [32,33]. However, this should not have a relevant influence on our results, primarily because the focus of this study focused on the difference between devices rather than absolute IO values, Moreover, all measurements were taken under the same conditions once sedation was effective within five minutes—an interval during which IOP is generally stable between minutes two and eight after anesthesia induction [33].

### Study Limitations

Despite its strengths, this study has several limitations that should be considered. First, the relatively small sample size and the retrospective design may limit the generalizability of the findings. A larger cohort would increase statistical power and allow for more robust conclusions. Second, the inclusion of both eyes from individual patients may have introduced bias by duplicating anatomical or physiological characteristics that could influence IOP measurements in a similar way across both eyes. Third, the lack of randomization in both the order of eye measurements (always starting with the right eye) and the sequence of tonometer use may have introduced order-related bias. Fourth, as all measurements were performed by the same examiner, masking was not possible. These limitations should be taken into account when interpreting the results and underline the need for future prospective studies with randomized protocols and larger, controlled populations. Another limitation is the lack of blinding during the measurement process. In this study, the investigator had access to prior IOP readings from different devices, which could have introduced observer bias. Future studies should implement blinded measurement protocols, where examiners are unaware of previous results, to ensure greater objectivity and eliminate potential bias in data collection.

The diversity of diagnoses among the included patients also presents a challenge. While the variability of diagnosis reflects real-world clinical practice, it also complicates direct comparisons between different tonometry methods. Future research could benefit from more uniform patient selection criteria, focusing on specific subgroups to improve the accuracy of device comparisons.

Additionally, since all IOP measurements were conducted under general anesthesia, the potential effects of anesthetic agents on IOP values must be considered. Although the study standardized the timing of IOP measurements to minimize these effects, it remains possible that anesthesia altered the IOP readings to some extent. Nevertheless, further investigation is needed to assess the potential impact of different anesthetic agents on IOP readings obtained with various tonometry devices [34,35]. Another factor that may have influenced the findings is corneal thickness variability among the study participants. The mean corneal pachymetry in this study was higher than in previous research, and it has been established in the literature that iCare tonometry tends to overestimate IOP in thicker corneas [36]. Since this study included patients with a higher-than-average corneal thickness, the degree of overestimation by iCare could have been more pronounced compared to other studies with thinner corneas. This should be taken into account when interpreting the results and when selecting a tonometer for pediatric patients with variable corneal properties.

Given these limitations, future research should focus on larger, prospective, multicenter studies including matched controls to improve statistical reliability and ensure a more diverse and representative patient population. Implementing blinded measurement protocols and ensuring a more homogeneous study cohort would further enhance the accuracy of findings. By addressing these limitations, future studies can provide a clearer and more definitive understanding of the performance of different tonometers in pediatric patients under general anesthesia.

## 5. Conclusions

This study aimed to compare Goldmann applanation tonometry (Perkins), iCare rebound tonometry, and the Tono-Pen in young children undergoing examination under general anesthesia. All patients were suspected of elevated IOP, making this a rare and clinically important study population.

Our findings suggest that the Perkins tonometer remains the preferred device in young patients under general anesthesia, particularly when IOP exceeds 25 mmHg or corneal thickness is abnormally high. For children with normal corneal thickness and IOP values equal or below 25 mmHg, the iCare tonometer proved to be a reliable alternative, offering ease of use and less operator dependency.

The Tono-Pen exhibited significant deviations, consistently overestimating IOP compared to Perkins, thus limiting its reliability in pediatric glaucoma evaluation under general anesthesia.

Overall, this study highlights the importance of selecting the most appropriate tonometry device for pediatric patients, particularly those requiring examination under anesthesia. Future research should aim to validate these findings in larger, multicenter trials, ensuring greater statistical power and improved generalizability.

## Figures and Tables

**Table 1:** Demographic and clinical characteristics of the study population.

**children (n)**	19
female (n)	11
male (n)	8
age (years)	1.8 ± 2.08
**eyes (n)**	38
aphakia (n)	18
congenital cataract	10
congenital glaucoma	7
nystagmus and/or hyperopia	3
**corneal diameter (mean ± SD; mm)**	horizontal: 10.6 ± 1.4 vertical: 10.1 ± 1.5
**corneal thickness (mean ± SD; µm)**	all eyes: 645 ± 135 aphakic eyes: 733 ± 267 phakic eyes: 604 ± 351 eyes with glaucoma: 744 ± 224
**IOP, all devices [mean ± SD, (range); mmHg]**	14.1 ± 7.4 (5–43)
Perkins	11.1 ± 4.8 (5–34)
iCare	11.6 ± 4.4 (6.4–36.4)
Tono-Pen	15.2 ± 5.5 (7–37)

**Table 2:** Bland–Altman regression analysis of the difference versus the mean for comparisons between Perkins, the Tono-Pen, and iCare. A significant positive slope indicates increasing disagreement with higher intraocular pressure (IOP). Results on the right show regressions after excluding IOP values >25 mmHg; none were statistically significant (*p* > 0.05).

Comparison Pair	Slope (All Values)	*p*-Value	Slope (IOP ≤ 25 mmHg)	*p*-Value
iCare vs. Perkins	0.42	<0.005	–0.12	0.39
Tono-Pen vs. Perkins	0.20	0.04	0.20	0.24
iCare vs. Tono-Pen	0.25	0.02	–0.30	0.15

## Data Availability

The raw data supporting the conclusions of this article will be made available by the authors on request.

## Supplementary Materials

The following supporting information can be downloaded at https://www.mdpi.com/article/10.3390/jcm14103338/s1. Figure S1. Scatterplots showing intraocular pressure (IOP) measurements of two tonometry devices for each study eye. Each point pair on the x-axis represents one study eye, with corresponding IOP values from the two compared devices plotted on the y-axis. As demonstrated by Bland-Altman analysis (Figure 1 and Figure 2), best agreement is observed between iCare and Perkins after excluding IOP values above 25 mmHg.
