# Supplementary material for: Comparison of Intraocular Pressure Measurements with Goldmann Applanation Tonometry, iCare, and Tono-Pen in Young Children with Anterior Segment Abnormalities Under General Anesthesia
Source: J Clin Med. 2025 May 11;14(10):3338. doi: 10.3390/jcm14103338 (PMC12111923; doi:10.3390/jcm14103338)
Supplement: Supplementary file 1 [file jcm-14-03338-s001.zip › jcm-3532215-supplementary.pdf]

Journal of Clinical Medicine

MDPI

1a

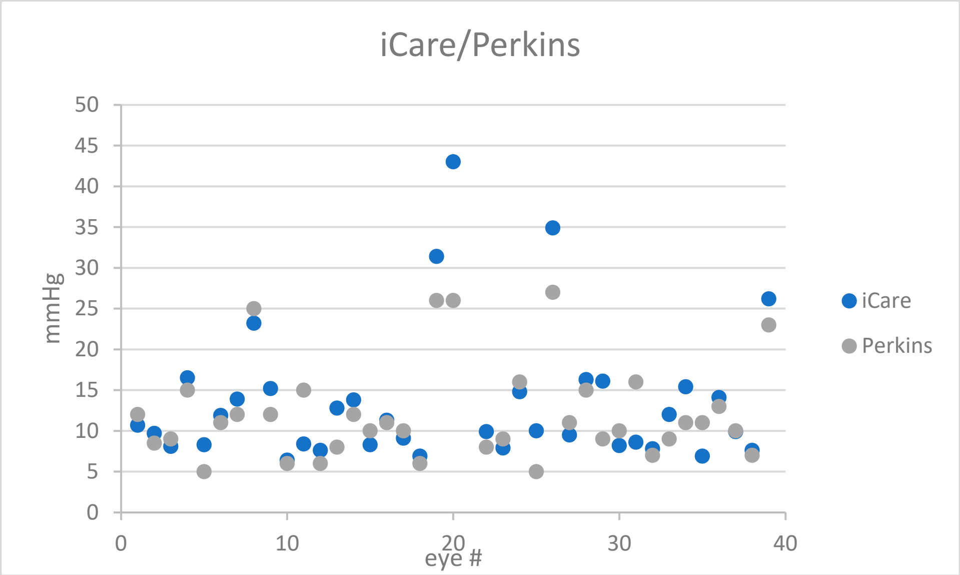

1b

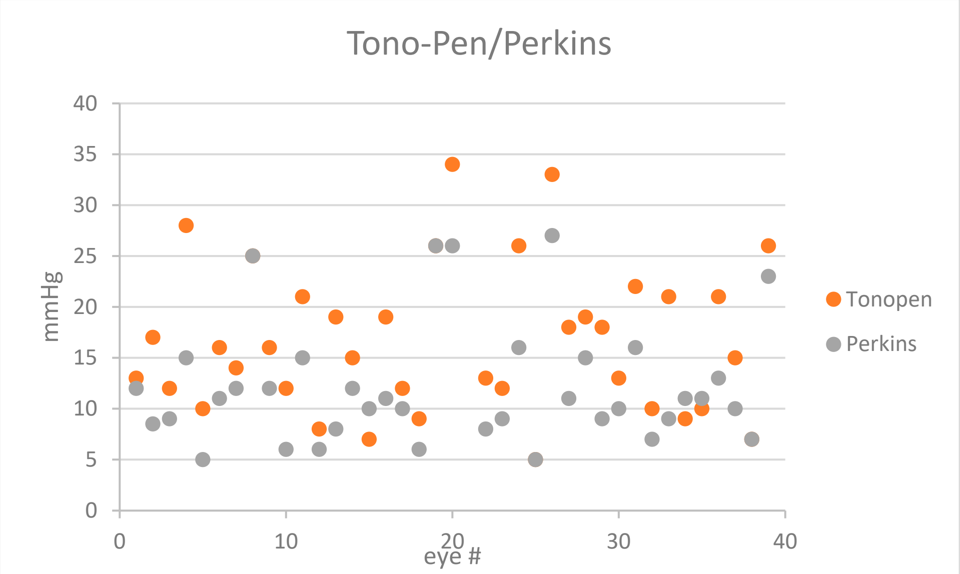

1c

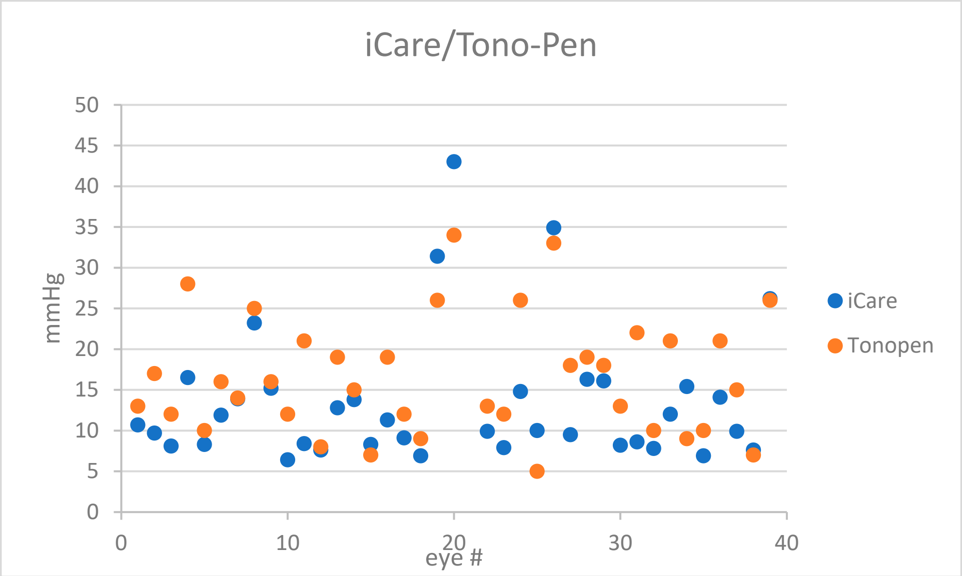

Figure S1. (**a–c**): Scatterplots showing intraocular pressure (IOP) measurements of two tonometry devices for each study eye. Each point pair on the x-axis represents one study eye, with corresponding IOP values from the two compared devices plotted on the y-axis. As demonstrated by Bland-Altman analysis (Figures 1 and 2), best agreement is observed between iCare and Perkins after excluding IOP values above 25 mmHg.
